# Psychological Burden Among Family Caregivers of People with Epilepsy in Limpopo and Mpumalanga Provinces, South Africa: A Qualitative Study

**DOI:** 10.3390/bs16071181

**Published:** 2026-07-13

**Authors:** Happiness Ngobeni, Lufuno Makhado, Thendo Gertie Makhado

**Affiliations:** 1 Department of Public Health, Faculty of Health Sciences, University of Venda, Thohoyandou 0950, South Africa; 11616209@mvula.univen.ac.za (H.N.); lufuno.makhado@univen.ac.za (L.M.); 2 Department of Advanced Nursing Sciences, Faculty of Health Sciences, University of Venda, Thohoyandou 0950, South Africa

**Keywords:** epilepsy, caregivers, psychological burden, qualitative research, South Africa, mental health

## Abstract

Epilepsy is a chronic neurological condition that often requires long-term care provided by family members, particularly in low-resource settings. This responsibility places caregivers at risk of significant psychological burden, which remains underexplored in rural South African contexts. This study explored the psychological burden experienced by family caregivers of people with epilepsy in Limpopo and Mpumalanga provinces, South Africa. A qualitative exploratory–descriptive design was employed. A total of 60 informal caregivers were snowball sampled from rural and peri-urban communities in Limpopo (*n* = 30) and Mpumalanga (*n* = 30). Data were collected through in-depth semi-structured interviews conducted in participants’ preferred languages. Interviews were audio-recorded, transcribed verbatim, and analysed using inductive thematic analysis. Three interrelated themes emerged, reflecting the psychological burden of caregiving: (1) persistent anxiety and fear related to unpredictable seizures, (2) emotional exhaustion and chronic stress, and (3) social isolation and psychological strain. Caregivers described constant worry, disrupted rest, and feelings of helplessness, often intensified by limited support and stigma associated with epilepsy. Caregivers of people with epilepsy experience a substantial psychological burden shaped by continuous caregiving demands and contextual challenges. There is a need for caregiver-focused mental health support and community-based interventions to reduce burden and improve well-being.

## 1. Introduction

Epilepsy is a chronic neurological disorder characterised by a predisposition to recurrent unprovoked seizures and is associated with biological, psychological, and social consequences for affected individuals and their families (Fisher et al., 2014). Although epilepsy can often be managed with appropriate diagnosis, treatment, and long-term support, many people with epilepsy continue to experience unpredictable seizures, stigma, social exclusion, and dependence on family members for daily care (Amjad et al., 2017; Arazeem et al., 2022). In low-resource and rural settings, where access to specialised neurological and mental health services may be limited, the responsibility for care frequently falls on family caregivers, who often provide unpaid, continuous, and emotionally demanding support (Ali et al., 2021; Muchada et al., 2021; Okiah et al., 2023).

Family caregivers of people with epilepsy may experience substantial psychological burden because of the unpredictable nature of seizures, fear of injury or death during seizure episodes, social stigma, financial strain, and uncertainty about how to respond effectively during emergencies (Yang et al., 2020; Samanta et al., 2022; X. Yu et al., 2025). Existing studies show that caregivers of people with epilepsy commonly report stress, anxiety, depression, emotional exhaustion, and reduced quality of life (Sabo et al., 2020; Yang et al., 2020; Cui et al., 2025). In South Africa, Sabo et al. (2020), found that caregiver burden among carers of children with epilepsy negatively affected health-related quality of life and family functioning. These findings suggest that epilepsy affects not only the diagnosed person but also the wider household and caregiving network.

The psychological burden experienced by caregivers may be shaped by clinical, social, and cultural factors. Seizure severity, seizure frequency, behavioural changes, and functional dependence may increase caregiver stress and emotional strain (Pokharel et al., 2020; Cui et al., 2025; X. Yu et al., 2025). At the same time, stigma and limited epilepsy-related knowledge can intensify fear, shame, secrecy, and social withdrawal (Amjad et al., 2017; Arazeem et al., 2022; Musekwa et al., 2023). In rural Limpopo and Mpumalanga, Musekwa et al. (2023) reported gaps in caregivers’ and family members’ knowledge, attitudes, and practices regarding epilepsy. Such knowledge gaps may contribute to anxiety during seizures and may delay appropriate help-seeking. Similarly, studies from rural South African communities have shown that epilepsy may be interpreted through cultural or traditional explanatory frameworks, which can influence treatment pathways, family responses, and community attitudes (Nemathaga et al., 2023).

Caregiver burden is also influenced by limited access to formal support systems. In many rural communities, caregivers may have little access to psychological intervention, psychoeducation, or community-based mental health services (Baker et al., 2026). As a result, family caregivers may rely mainly on personal coping strategies, relatives, neighbours, religious support, or traditional/community structures. However, when support is inadequate, caregivers may experience isolation, helplessness, chronic stress, and emotional fatigue (Udoh et al., 2021; Nema et al., 2025; Poprelka et al., 2025). Evidence from systematic and critical reviews further shows that caregiver burden in epilepsy is multidimensional, involving psychological, social, economic, and practical challenges that require context-specific interventions (Poprelka et al., 2025; X. Yu et al., 2025).

Recent qualitative and review studies have shown that caregivers of people with epilepsy experience substantial psychological and social burden associated with long-term caregiving demands. Caregiving is influenced by seizure unpredictability, continuous caregiving responsibilities, and limited support, all of which contribute to anxiety, emotional exhaustion, stigma, social isolation, financial strain, and persistent concern for the well-being of the person living with epilepsy (Z. Yu et al., 2022; X. Yu et al., 2023; Okiah et al., 2023). However, existing literature remains largely descriptive and provides limited conceptual understanding of how caregiver burden develops within broader social, cultural, and healthcare contexts (Breed et al., 2025; Poprelka et al., 2025). Emerging evidence suggests that caregiver distress results from interconnected stressors such as stigma, financial hardship, inadequate healthcare support, and ongoing caregiving responsibilities, particularly in low-resource rural settings (Carter et al., 2022; Wang et al., 2024).

Drawing on the Stress Process Model proposed by Pearlin et al. (1990), psychological burden among caregivers can be understood as a process that develops over time because of ongoing caregiving demands. The model explains how primary stressors, such as managing unpredictable seizures, monitoring treatment adherence, and responding to seizure-related emergencies, may interact with secondary stressors, including financial strain, social isolation, stigma, disrupted family roles, and reduced employment opportunities. These stressors may accumulate and negatively affect caregivers’ emotional well-being and coping capacity. The model further emphasises that caregiver experiences are influenced by broader social and structural conditions, including access to healthcare services, availability of social support, socioeconomic circumstances, and cultural perceptions surrounding illness. Within the context of epilepsy care in rural South Africa, where epilepsy-related stigma and limited healthcare resources remain significant challenges, the Stress Process Model provides a useful framework for understanding how caregiving responsibilities evolve into sustained psychological distress. Applying this conceptual lens may therefore help move beyond descriptive accounts of caregiver experiences towards a deeper understanding of the underlying social and contextual processes shaping caregiver burden. Using this framework enables a deeper understanding of how caregiving demands interact with the rural context to shape caregivers’ psychological burden, rather than simply describing caregivers’ experiences.

Previous qualitative studies have consistently reported anxiety, emotional exhaustion, stigma, and social isolation among caregivers of people with epilepsy (Amjad et al., 2017; Okiah et al., 2023; Z. Yu et al., 2022; X. Yu et al., 2023). However, there remains limited qualitative evidence from rural South African settings exploring how these psychological experiences are shaped by the interaction of caregiving demands with broader structural and sociocultural factors, including poverty, limited access to healthcare, epilepsy-related stigma, and cultural beliefs. Guided by Pearlin’s Stress Process Model, this study explores the psychological burden experienced by family caregivers of people with epilepsy in Limpopo and Mpumalanga provinces, South Africa. By examining caregivers’ lived experiences through this theoretical lens, the study extends existing qualitative research by demonstrating how primary caregiving stressors interact with contextual stressors to produce sustained psychological burden in low-resource rural settings.

## 2. Methods

This study employed a qualitative, exploratory–descriptive design to explore the psychological burden experienced by family caregivers of people with epilepsy. A qualitative approach was appropriate as it enabled an in-depth understanding of caregivers’ lived experiences, emotional challenges, and the mental strain associated with caregiving (M. Hennink et al., 2020; Okiah et al., 2023). The study was conducted in rural and peri-urban communities in Limpopo and Mpumalanga provinces of South Africa. Specifically, the study sites included Collins Chabane Municipality in the Vhembe District (Limpopo) and Bushbuckridge in the Ehlanzeni District (Mpumalanga). These settings are characterised by high levels of poverty, limited healthcare infrastructure, long distances to health facilities, and strong cultural belief systems that may influence perceptions and management of epilepsy (Wagner et al., 2020; Makhado et al., 2024). These contextual factors made the settings particularly relevant for exploring caregiving experiences and psychological burden.

### 2.1. Population and Sampling

The study population consisted of informal caregivers of people with epilepsy. For this study, an informal caregiver was defined as an adult family member or relative who provided primary or substantial unpaid care to a person with a clinical diagnosis of epilepsy within the same household. Participants included parents, grandparents, spouses, siblings, and other relatives, including extended family members such as aunts, in-laws, cousins and uncles involved in daily caregiving. A purposive sampling strategy, complemented by snowball sampling, was employed in this study, as caregivers are not always easily identifiable through formal records, particularly in rural settings (Noy, 2008). Participants who satisfied the inclusion criteria were initially identified through community engagement activities and existing local networks. These caregivers were then asked to recommend other individuals who met the study requirements, thereby facilitating recruitment through snowball sampling. Eligibility criteria required participants to be at least 18 years old, reside within the study area, and be actively involved in providing ongoing care to a family member living with epilepsy. Individuals who were unable to provide written informed consent or who were not directly engaged in caregiving were excluded from participation. Recruitment and data collection continued until data saturation was attained, meaning that additional interviews no longer generated new themes, perspectives, or relevant insights (M. M. Hennink et al., 2017; Guest et al., 2020). Table S1 (Supplementary Material S1) provides an overview of the participants’ sociodemographic characteristics.

### 2.2. Data Collection Procedure

Data were collected through in-depth semi-structured interviews conducted between December 2025 and February 2026. Data were collected using a semi-structured interview guide developed from the study objectives and relevant literature (Supplementary Material S2). This approach enabled participants to share detailed accounts of their caregiving experiences while ensuring that key topics related to psychological burden were covered (DeJonckheere & Vaughn, 2019). The interview guide covered caregivers’ daily caregiving responsibilities, the emotional and physical demands associated with caring for a family member with epilepsy, community and cultural perceptions of epilepsy, caregiving challenges and facilitators, experiences of stigma and discrimination, available support systems, coping strategies, and perceptions of community attitudes towards people living with epilepsy and their families. Participants were also invited to reflect on how these experiences affected their well-being and family life. Throughout the interviews, probing questions were used to encourage participants to expand on their responses, clarify their views, provide examples, and explore issues in greater depth, thereby generating richer and more nuanced data (Osborne & Grant-Smith, 2021). Interviews were conducted by the researcher (H.N.) in participants’ preferred languages, including Xitsonga, Tshivenda, Sepedi, and siSwati. Interviews took place in private and convenient locations, such as participants’ homes or other mutually agreed safe settings, and lasted approximately 40 to 65 min. With participants’ consent, interviews were audio-recorded and supplemented with field notes documenting non-verbal cues, contextual observations, and preliminary reflections. Data collection and analysis occurred concurrently, with transcripts reviewed after each interview to identify emerging patterns and themes. Data saturation was considered to have been reached when repeated patterns and experiences were consistently observed across participants from both provinces and subsequent interviews yielded no substantially new information relevant to the study objectives. Although saturation had been achieved, recruitment continued across different communities in Limpopo and Mpumalanga to ensure that the findings reflected experiences beyond individual caregiver networks, given the use of snowball sampling, and to allow comparison of caregiving experiences across the two study settings. The additional interviews confirmed the consistency of the identified themes rather than generating new ones. The final sample comprised 60 family caregivers, including 30 participants from Limpopo Province and 30 from Mpumalanga Province. In Mpumalanga, 20 participants were female and 10 were male, while in Limpopo, 26 participants were female and 4 were male, reflecting the predominance of women in caregiving roles within the study settings.

Audio recordings were transcribed verbatim, and interviews conducted in local languages were translated into English by bilingual members of the research team. To ensure accuracy and preserve the original meaning of participants’ accounts, translated transcripts were cross-checked against the audio recordings and the original-language transcripts. During data analysis, careful attention was given to culturally specific words, expressions, and descriptions of emotional distress used by participants. Where there was no direct English equivalent, bilingual members of the research team discussed the meaning within its cultural context until consensus was reached on the most appropriate interpretation. This process ensured that coding was guided by the intended conceptual meaning of participants’ narratives rather than by literal word-for-word translation. The research team also collaboratively reviewed the translated transcripts to verify consistency, resolve translation discrepancies, and ensure that participants’ intended meanings and contextual expressions were retained throughout the analysis.

### 2.3. Data Analysis

Data were analysed using inductive thematic analysis. This approach allowed for the identification and interpretation of patterns related to psychological burden across participants’ narratives while remaining grounded in their experiences (Braun & Clarke, 2006). Although themes were generated inductively from participants’ narratives, Pearlin’s Stress Process Model was subsequently used as an interpretive framework to examine how the identified themes reflected the interaction between primary caregiving stressors, secondary stressors, and their consequences for caregivers’ psychological well-being. This approach enabled the analysis to remain grounded in participants’ lived experiences while providing a theoretical explanation of how psychological burden develops within the rural South African context. The analysis followed six steps (Braun & Clarke, 2006). First, the researchers familiarised themselves with the data by reading and re-reading transcripts. Second, initial codes were generated from meaningful segments of the data. Third, similar codes were grouped into categories and potential themes. Fourth, themes were reviewed and refined in relation to the dataset. Fifth, themes were clearly defined and named. Finally, relevant verbatim quotations were selected to support each theme. ATLAS.ti Version 8.4 was used to organise and manage the data. To enhance the reliability and consistency of the analysis, the primary researcher conducted the initial coding of the transcripts, after which supervisors independently reviewed selected transcripts and emerging codes. An independent co-coder with qualitative research experience was also engaged to review the coding process and preliminary themes. Thereafter, meetings were held between the primary researcher, research team members, and the independent co-coder to compare codes, discuss similarities and differences in interpretation, resolve discrepancies, and refine the development of themes and subthemes. Consensus was reached through collaborative discussion to ensure that the themes accurately reflected participants’ experiences and the meanings embedded within the data. Trustworthiness was ensured using the criteria of credibility, dependability, confirmability, and transferability (Lincoln & Guba, 1985). Credibility was strengthened through prolonged engagement with the data, the use of verbatim quotations, and regular discussions among the research team (Nowell et al., 2017). Where applicable, member checking was conducted by clarifying issues during interviews or sharing summaries with selected participants (Birt et al., 2016). Dependability and confirmability were enhanced through maintaining an audit trail, reflexive note-taking, and collaborative coding discussions among the research team to ensure consistency and reduce researcher bias (Lincoln & Guba, 1985; Nowell et al., 2017). Transferability was addressed by providing detailed descriptions of the study context and participant characteristics to allow readers to assess the applicability of the findings to other settings (Lincoln & Guba, 1985).

### 2.4. Ethical Considerations

Ethical approval was obtained from the University of Venda Human and Clinical Trials Research Ethics Committee (HCTREC) (Ethical Clearance No. FHS/25/PH/22/1811). Permission to conduct the study was also obtained from relevant traditional authorities and community leaders. Participation was voluntary, and written informed consent was obtained from all participants before data collection. Participants were informed about the purpose of the study, their right to decline participation, and their right to withdraw at any time without any consequences. Confidentiality and anonymity were ensured by removing identifying information from transcripts and using pseudonyms or participant codes. All data, including audio recordings, transcripts, and field notes, were stored securely and were accessible only to the research team.

## 3. Results

### 3.1. Theme 1: Persistent Anxiety and Fear Related to Unpredictable Seizures

Caregivers across both Limpopo and Mpumalanga described epilepsy as an unpredictable and emotionally distressing condition characterised by sudden and often traumatic seizure episodes. The unpredictability of seizures emerged as a major source of ongoing psychological distress, with caregivers describing constant fear, hypervigilance, and anxiety about the possibility of seizures occurring unexpectedly. Participants explained that seizures could happen at any time and in any setting, creating a persistent sense of uncertainty and emotional instability within the caregiving experience.

In Limpopo, caregivers described intense fear and shock during seizure episodes, particularly when seizures involved collapse, foaming at the mouth, loss of consciousness, or body shaking. These experiences were often perceived as frightening and life-threatening, especially during initial seizure episodes when caregivers lacked knowledge and understanding of epilepsy. One participant recounted the emotional distress experienced after unexpectedly witnessing a seizure episode:

*“It was just a normal day and we were preparing to go to the shops that so I don’t know if it was because it was hot day we shocked to find her on the kitchen floor shaking and foam was coming out of her mouth. …”*

(Participant 21, Limpopo)

Similarly, caregivers from Mpumalanga described seizure episodes as emotionally traumatic experiences associated with panic, helplessness, and fear of death. Participants explained that witnessing unresponsiveness, body weakness, or loss of bodily control during seizures often led them to believe that the person had died. One caregiver described the emotional impact of witnessing such an episode:

*“It was a very painful day because I just heard “mhmm” mhmm sounds, and when I went to check, he was already on the floor. He was cold and not responding. We were so sure that he had died because he had already excreted on himself.”*

(Participant 18, Mpumalanga)

The findings further revealed that fear extended beyond seizure episodes themselves and became embedded within caregivers’ everyday lives. Many participants described living in a constant state of alertness due to uncertainty about when the next seizure might occur. Routine daily activities were frequently interrupted by ongoing monitoring and supervision of the person living with epilepsy to prevent potential injuries during seizures. This continuous vigilance contributed significantly to psychological strain and emotional fatigue. As explained by caregivers from Mpumalanga:

*“You always must observe them the whole day so that they cannot get hurt.”*

(Participant 2, Mpumalanga)

*“You can’t even take an hour without seeing them. You have to make sure you are always here with them.”*

(Participant 3, Mpumalanga)

Across both provinces, caregivers also reflected on the emotional trauma associated with first-time seizure experiences. Participants described feelings of confusion, panic, helplessness, and fear during the initial onset of epilepsy, particularly due to limited knowledge regarding seizure management and emergency responses. In some cases, first seizure experiences resulted in emergency hospitalisation and heightened fears about survival and long-term health outcomes. Within Pearlin’s Stress Process Model, the experiences described in this theme represent primary caregiving stressors arising directly from caring for a person with epilepsy. Unpredictable seizures, constant supervision, emergency preparedness, and responsibility for preventing injury required caregivers to remain continuously vigilant. These ongoing caregiving demands formed the foundation of the stress process and contributed to persistent anxiety and emotional strain. The findings show that seizure unpredictability was not simply a source of fear but the primary stressor from which caregivers’ psychological burden began to develop.

### 3.2. Theme 2: Emotional Exhaustion and Chronic Stress

Caregivers across both Limpopo and Mpumalanga described caregiving as emotionally demanding and psychologically draining. Participants reported experiencing ongoing emotional exhaustion, chronic stress, sadness, helplessness, and emotional instability resulting from the continuous responsibility of caring for a person living with epilepsy. The long-term nature of caregiving, combined with persistent fear of seizures and limited opportunities for rest, contributed to sustained psychological strain among caregivers.

In Limpopo, emotional distress was strongly associated with the prolonged caregiving burden and feelings of helplessness in managing epilepsy-related challenges. Some caregivers expressed frustration and emotional pain arising from their inability to prevent seizures or improve the condition of their loved ones. One participant explained:

*“The stress just torments you. What can you do?”*

(Participant 15, Limpopo)

This statement reflected the sense of emotional defeat and psychological burden experienced by many caregivers who felt overwhelmed by the unpredictable and chronic nature of epilepsy. Participants described caregiving as emotionally consuming, with some indicating that they constantly worried about the health, safety, and future of the person living with epilepsy.

Similarly, caregivers from Mpumalanga described emotionally distressing experiences during severe seizure episodes, particularly when seizures resulted in injuries, prolonged unconsciousness, or hospitalisation. Witnessing such events often triggered intense emotional reactions, including panic, fear, crying, and emotional breakdowns. One caregiver recalled the emotional trauma experienced after receiving news of a seizure incident at school:

*“There was a day when he started falling at school. They told me that he didn’t fall in a good way, he hit the desk with his head and was unconscious for 5 h. Upon my arrival at the school, I started crying, and my hands were on my head. I cried so badly that day.”*

(Participant 26, Mpumalanga)

Caregivers further described emotional exhaustion associated with behavioural, cognitive, and personality changes observed in persons living with epilepsy. In Mpumalanga, some participants explained that seizures were followed by confusion, hallucinations, dependency, and altered behaviour, which increased emotional strain and feelings of grief and uncertainty. One caregiver explained:

*“It was the last time he had seizures, and after that he started having illusions and saying things that aren’t there. That was so hard because I didn’t know if I was losing him bit by bit.”*

(Participant 13, Mpumalanga)

This finding suggested that caregivers were not only burdened by the physical management of seizures but were also emotionally affected by perceived changes in the identity, functioning, and independence of the person living with epilepsy. The findings additionally revealed that emotional burden was intensified by continuous caregiving responsibilities and lack of rest. Caregivers described disrupted sleep, night-time monitoring, and the need to remain constantly available in case seizures occurred unexpectedly. Many participants indicated that caregiving responsibilities extended throughout the day and night, leaving little opportunity for emotional recovery or self-care. Over time, this persistent responsibility contributed to chronic stress, emotional fatigue, and psychological exhaustion.

*“We manage because always keep an eye on her and I’m not the only one caring for her we help each other as a family. We always check on her even at night when she is sleeping.”*

(Participant 19, Limpopo)

*“So, knock shift is when you work during the night. So a patient can fall during the night. So it is good to listen and to always be vigilant.”*

(Participant 30, Mpumalanga)

Caregivers across both provinces experienced caregiving as an emotionally overwhelming process characterised by ongoing stress, helplessness, emotional trauma, and exhaustion. The cumulative emotional demands associated with epilepsy caregiving significantly affected caregivers’ psychological well-being and daily functioning. Within Pearlin’s Stress Process Model, emotional exhaustion observed in this theme represents the psychological consequences of prolonged exposure to primary caregiving stressors. Continuous seizure monitoring, disrupted sleep, emergency caregiving, and uncertainty regarding future seizures progressively depleted caregivers’ emotional resources. These sustained demands reduced caregivers’ capacity for emotional recovery and contributed to chronic stress, making them increasingly vulnerable to persistent psychological distress.

### 3.3. Theme 3: Social Isolation and Psychological Strain

Caregivers across both Limpopo and Mpumalanga described caregiving as socially restrictive and emotionally isolating. Participants explained that the continuous responsibility of monitoring and caring for a person living with epilepsy significantly affected their social lives, employment opportunities, community participation, and interpersonal relationships. Social isolation emerged as both a consequence of caregiving demands and a contributor to ongoing psychological distress.

In Limpopo, caregivers frequently described being unable to participate in normal social and economic activities because they could not leave the person living with epilepsy unattended. Participants explained that constant supervision was necessary due to fear of sudden seizures and possible injuries. As a result, some caregivers reported sacrificing employment opportunities, social interaction, and personal freedom in order to fulfil caregiving responsibilities. One participant explained:

*“My challenge is that I can’t go anywhere, I can’t get away from her, and I can’t even go to work. I have to stay at home and look after her. It is a challenge because I can’t go to work and leave her all alone the whole day.”*

(Participant 23, Limpopo)

Similarly, caregivers from Mpumalanga described restricted movement and withdrawal from social activities because of concerns about leaving the person with epilepsy alone at home. Participants expressed feelings of frustration and emotional burden associated with being constantly tied to caregiving responsibilities. One caregiver stated:

*“Eish, I can’t do much because I have no one to leave him with. I can’t even go to work.”*

(Participant 1, Mpumalanga)

The findings further revealed that social isolation was reinforced by epilepsy-related stigma and fear of public seizure episodes. Caregivers described feeling anxious and embarrassed about how community members might react if seizures occurred in public settings. This fear contributed to avoidance of social gatherings, public spaces, and community events, resulting in reduced social engagement and increased emotional withdrawal. Some participants explained that community members lacked understanding of epilepsy, which intensified feelings of exclusion and emotional discomfort.

*“Most people think it’s witchcraft. I have never uhm… I remember there was a time we visited the clinic, and most people started to move away from us when the person with epilepsy had an episode. It was only a few people who came to help us, but after that, we were able to get urgent help and jump the queue to see the doctor. Some thought it was witchcraft, you see?”*

(Participant 1, Mpumalanga)

Within Pearlin’s Stress Process Model, the experiences described in this theme represent secondary stressors that emerged alongside ongoing caregiving responsibilities. Social isolation, employment disruption, financial strain, altered family roles, and epilepsy-related stigma developed as consequences of prolonged caregiving demands and further contributed to caregivers’ psychological strain. These findings demonstrate that caregiver burden extended beyond the direct demands of epilepsy care to include broader social and contextual challenges experienced by caregivers.

## 4. Discussion

This study explored the psychological burden experienced by family caregivers of persons with epilepsy (PWE) in Limpopo and Mpumalanga provinces, South Africa. Across both provinces, caregiving was characterised by persistent anxiety, emotional exhaustion, and social isolation, largely driven by the unpredictable nature of seizures, continuous caregiving demands, and limited psychosocial support. These findings support existing evidence that caregiving in epilepsy is a prolonged and psychologically demanding experience that significantly affects caregivers’ emotional well-being and quality of life (Pokharel et al., 2020; X. Yu et al., 2023).

Caregivers described seizures as sudden, traumatic, and potentially life-threatening events that created continuous uncertainty and emotional distress. Similar findings have been reported in qualitative studies from Uganda and Malaysia, where caregivers described living in constant fear of seizure recurrence, injury, or death, resulting in heightened vigilance and chronic anxiety (Okiah et al., 2023). The present findings also align with those of (Pokharel et al., 2020), who identified seizure unpredictability as a significant predictor of caregiver burden and psychological distress. Caregivers in the current study frequently described the need to constantly monitor the person with epilepsy, even during routine daily activities and at night, reflecting what previous literature has conceptualised as “hypervigilant caregiving” (Penovich et al., 2021). Such hypervigilance may contribute to emotional fatigue, chronic stress, and impaired psychological recovery over time.

The emotional reactions described by participants, including panic, helplessness, fear of death, and traumatic responses during initial seizure episodes, further highlight the profound psychological impact of epilepsy caregiving. Caregivers often interpreted first seizures as medical emergencies or fatal events, particularly when accompanied by loss of consciousness, foaming at the mouth, or bodily collapse. Similarly, Z. Yu et al. (2022) reported that caregivers frequently experience intense emotional trauma during initial seizure episodes because of limited epilepsy knowledge and uncertainty regarding seizure management. In low-resource settings, where epilepsy education and psychosocial counselling may be limited, such experiences can intensify fear, confusion, and long-term anxiety among caregivers (Muchada et al., 2021). These findings reinforce the importance of caregiver education programmes aimed at improving seizure recognition, emergency response knowledge, and emotional preparedness.

Participants described feelings of helplessness, sadness, emotional breakdown, and emotional instability resulting from continuous caregiving demands and prolonged exposure to seizure-related crises. This supports previous studies demonstrating that caregiver burden in epilepsy extends beyond physical caregiving responsibilities and includes significant emotional and psychological strain (Cui et al., 2025; Poprelka et al., 2025; X. Yu et al., 2025). Research conducted by Cui et al. (2025) also reported elevated levels of psychological distress, depression, and emotional burden among caregivers of adults with epilepsy. Likewise, Z. Yu et al. (2022) found that caregivers frequently experience emotional exhaustion due to constant worry about seizures, uncertainty regarding the future, and ongoing caregiving responsibilities.

An important finding in the present study was caregivers’ distress associated with behavioural, cognitive, and personality changes observed in persons with epilepsy. Some caregivers described feelings of grief and emotional pain as they perceived gradual changes in the identity and functioning of their loved ones following recurrent seizures. This finding reflects what has been described in caregiving literature as anticipatory grief, where caregivers experience emotional distress linked to perceived progressive loss and altered interpersonal relationships (Z. Yu et al., 2022). Such experiences may be particularly distressing in epilepsy caregiving because caregivers often witness unpredictable fluctuations in cognitive functioning, behaviour, and social independence. The emotional impact of these changes further contributes to psychological strain and feelings of helplessness among caregivers.

The study also revealed that chronic caregiving responsibilities disrupted caregivers’ rest and recovery. Participants described sleep disruption, night-time monitoring, and continuous alertness due to fear of nocturnal seizures and seizure-related injuries. Sleep deprivation and prolonged caregiving vigilance have been associated with caregiver burnout, emotional exhaustion, and poor mental health outcomes across chronic illness caregiving contexts (Pawl et al., 2013). Continuous caregiving without adequate opportunities for respite may therefore reinforce a cycle of chronic stress and emotional fatigue. Within the framework of the Stress Process Model (Pearlin et al., 1990). These findings suggest that ongoing exposure to caregiving stressors without sufficient coping resources or social support may progressively compromise caregivers’ psychological well-being over time.

This study also found that there was significant social isolation experienced by caregivers. Participants reported withdrawal from employment, social activities, community participation, and interpersonal relationships because of the continuous need to supervise and care for the person with epilepsy. Similar findings have been reported in previous studies where caregivers described caregiving as socially restrictive and emotionally isolating (Okiah et al., 2023; X. Yu et al., 2023). In many cases, caregivers sacrificed employment opportunities and social engagement due to fears that seizures could occur unexpectedly in their absence. This social withdrawal not only reduced caregivers’ social support networks but also intensified feelings of loneliness, stress, and emotional burden.

Stigma further emerged as a major contributor to psychological strain and social isolation. Caregivers described embarrassment, fear of public reactions, and avoidance of social spaces because of negative community attitudes towards epilepsy. These findings are consistent with studies demonstrating that epilepsy-related stigma affects not only persons with epilepsy but also their family members and caregivers (Amjad et al., 2017; Arazeem et al., 2022). In African contexts, epilepsy is still frequently associated with witchcraft, spiritual causes, or contagion, contributing to discrimination, social exclusion, and delayed healthcare-seeking behaviour (Musekwa et al., 2023). The findings from this study suggest that stigma continues to shape caregiving experiences in rural South African communities, reinforcing emotional distress and limiting caregivers’ willingness to seek social support.

The rural context appeared to shape caregivers’ experiences in several important ways. Limited access to specialised neurological services, long travel distances to healthcare facilities, financial hardship, and persistent cultural beliefs surrounding epilepsy reduced opportunities for caregivers to access both practical and emotional support. Consequently, caregivers often relied on family members and personal coping strategies despite the considerable demands of caregiving. These contextual factors may explain why participants described sustained psychological distress rather than temporary periods of stress. Compared with caregivers in higher-resource settings, where multidisciplinary epilepsy services, psychosocial support, and temporary caregiving support allow caregivers time to rest, in rural South Africa, caregivers appeared to shoulder a greater proportion of caregiving responsibilities with limited formal support.

The interconnectedness between social isolation and psychological distress was also evident in the findings. Caregivers who lacked adequate emotional, familial, or community support appeared more vulnerable to psychological distress. Existing literature consistently identifies social support as a protective factor that may buffer caregiver burden and improve coping capacity (Düken & Belli, 2025). However, in contexts characterised by stigma, poverty, and limited access to formal mental health services, caregivers may have limited opportunities to access supportive networks or psychosocial interventions. This highlights the importance of community-based caregiver support programmes, peer support groups, and stigma reduction interventions within epilepsy care services.

**Figure 1:**
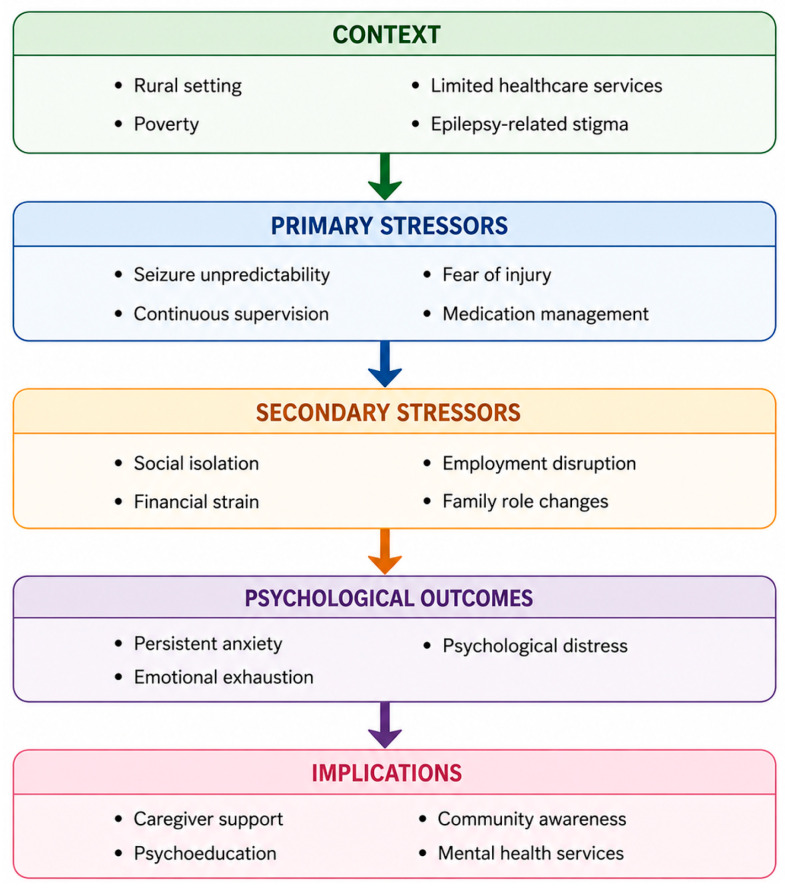
Application of Pearlin’s Stress Process Model to Family Caregivers of People with Epilepsy in Rural South Africa.

Guided by Pearlin’s Stress Process (Pearlin et al., 1990), the findings of this study demonstrate that caregiver burden developed through a cumulative stress process rather than as isolated psychological experiences. The relationships among these stressors and their cumulative effects are illustrated in Figure 1. Persistent anxiety and fear arose from primary caregiving stressors, including seizure unpredictability, continuous supervision, emergency caregiving, and medication management. Over time, these demands contributed to emotional exhaustion and were further compounded by secondary stressors, including social isolation, employment disruption, financial strain, and epilepsy-related stigma, resulting in sustained psychological burden. Research from Uganda, Malaysia, and China, indicated that caregivers described living in persistent fear of seizure recurrence, injury, or death, resulting in ongoing psychological strain and hypervigilance (Yang et al., 2020; Okiah et al., 2023; X. Yu et al., 2023). However, the present study extends existing evidence by demonstrating how these primary caregiving stressors are further intensified within rural South African communities by contextual factors. These structural and social conditions amplify the stress process by increasing caregivers’ sense of responsibility while limiting access to resources that could buffer psychological distress. This finding underscores the importance of considering both caregiving demands and the broader sociocultural context when developing interventions aimed at improving caregiver well-being. These interconnected stressors, operating within a context of limited healthcare resources and poverty, contributed to persistent anxiety, emotional exhaustion, and psychological distress. This interpretation extends previous qualitative studies by demonstrating how caregiver burden develops through dynamic interactions between caregiving demands and contextual factors in rural South African communities.

The similarities observed across both provinces suggest that the psychological burden experienced by caregivers may reflect broader systemic and contextual challenges affecting epilepsy care in low-resource South African settings. Interventions should therefore move beyond patient-centred seizure management and incorporate caregiver-focused psychosocial support, epilepsy education, counselling services, and community-based stigma reduction initiatives. Strengthening caregiver support systems may not only improve caregiver well-being but may also positively influence the quality of care provided to persons with epilepsy.

## 5. Limitations

Despite its contributions, the study has several limitations. As a qualitative study with a relatively small, snowball-selected sample, the findings are not generalisable to all caregivers of people with epilepsy in South Africa. Additionally, the reliance on self-reported experiences may introduce recall bias or social desirability bias, particularly when discussing emotionally sensitive topics. Furthermore, the study did not include perspectives of healthcare providers or the individuals with epilepsy themselves, which may have provided a more comprehensive understanding of caregiving dynamics. Moreover, contextual factors such as socioeconomic status and access to healthcare services were not explored in depth, which may influence caregiving experiences. The predominance of female participants reflects the gendered nature of caregiving within the study communities. However, the relatively small number of male caregivers may have limited the exploration of male caregiving experiences and perspectives. Future studies should purposefully recruit more male caregivers to better understand potential gender differences in caregiving experiences. In conclusion, language translation during data collection and analysis may have resulted in a slight loss of meaning.

## 6. Implications and Recommendations

The findings of this study have important implications for clinical practice, public health, and policy. Healthcare providers should routinely assess the psychological well-being of family caregivers during epilepsy follow-up visits and provide caregiver-focused psychoeducation on seizure recognition, seizure first aid, medication management, and coping strategies. Counselling services and caregiver support groups should also be integrated into epilepsy care to help reduce anxiety, emotional exhaustion, and social isolation.

At the community level, awareness campaigns should be implemented to improve public understanding of epilepsy and reduce stigma and misconceptions, particularly in rural communities where epilepsy is often associated with witchcraft or other traditional beliefs. Community health workers and primary healthcare clinics could play an important role in providing caregiver education, home-based follow-up, and referral for psychosocial support. Collaborating with community leaders, traditional leaders, and faith-based organisations may also help promote accurate information about epilepsy and encourage community acceptance of people with epilepsy and their caregivers.

At the policy level, epilepsy care programmes should recognise family caregivers as essential partners in patient care and include caregiver support within routine epilepsy services. Policymakers should strengthen access to community-based mental health services in rural areas and consider introducing respite care services, where feasible, to provide temporary relief for caregivers with high caregiving demands. Greater investment in training primary healthcare workers on caregiver support and epilepsy management would further improve the quality of care for both caregivers and people with epilepsy.

Future research should explore caregivers’ experiences over time to better understand how psychological burden develops throughout the caregiving journey. Studies should also evaluate the effectiveness of caregiver support interventions, particularly community-based psychoeducation programmes, peer support groups, and stigma reduction initiatives. The predominance of female participants reflects the gendered nature of caregiving within the study communities. However, the relatively small number of male caregivers may have limited the exploration of male caregiving experiences and perspectives. Consequently, the findings may more strongly reflect the psychological burden experienced by female caregivers. Future studies should purposefully recruit more male caregivers to better understand potential gender differences in caregiving experiences and support needs.

## 7. Conclusions

This study highlights the substantial psychological burden experienced by family caregivers of people with epilepsy in Limpopo and Mpumalanga provinces. Caregiving is characterised by persistent anxiety due to seizure unpredictability, chronic emotional exhaustion, and significant social isolation. These interconnected challenges negatively impact caregivers’ mental health and overall well-being. The findings underscore the urgent need for comprehensive, caregiver-inclusive approaches to epilepsy management that extend beyond clinical treatment of seizures. Addressing caregiver burden through psychosocial support, education, and community engagement is critical to improving both caregiver well-being and the quality of care provided to PWE.

## Data Availability

The data presented in this study are not publicly available due to ethical and confidentiality considerations associated with qualitative interview data. Data may be available from the corresponding author upon reasonable request and subject to ethical approval.

## Supplementary Materials

The following supporting information can be downloaded at: https://www.mdpi.com/article/10.3390/bs16071181/s1, Supplementary Material S1: Demographic Characteristics of Participants; Supplementary Material S2: Semi-Structured Interview Guide for Caregivers of People with Epilepsy.
