# Supplementary material for: Psychological Burden Among Family Caregivers of People with Epilepsy in Limpopo and Mpumalanga Provinces, South Africa: A Qualitative Study
Source: Behav Sci (Basel). 2026 Jul 13;16(7):1181. doi: 10.3390/bs16071181 (PMC13405876; doi:10.3390/bs16071181)
Supplement: Supplementary file 1 [file behavsci-16-01181-s001.zip › SUPPLEMENTARY TABLE S1.pdf]

Supplementary Table S1: Demographic Characteristics of Participants

CHARACTERISTIC	MPUMALANGA	LIMPOPO	TOTAL
NUMBER OF PARTICIPANTS	30	30	60
GENDER			
**MALE**	10	4	14
**FEMALE**	20	26	46
AGE	≥18 years	≥18 years	≥18 years
CAREGIVER INCLUSION CRITERION	Minimum 6 months of caregiving experience	Minimum 6 months of caregiving experience	—
PREDOMINANT CAREGIVING DURATION	Majority >3 years	Majority >3 years	Majority >3 years
